# 

**Published:** 1885-06

**Authors:** 


CONTENTS

Vol. 32 No. 6

Why Alcohol Intoxicates... 1
Ginseng... 2
A Dozen Hardy Shrubs... 3
Out Door Life for Women... 4
Disinfection and Disinfectants... 5
Liberty Enlightening the World... 10
The Faith Cure Folly... 11
Origin of "Humbug"... 13
Scarlet Fever... 15
Elecampane as an Antiseptic... 16
Ventilation... 19
Effect of Alcohol on the Arteries... 20

